# Artificial intelligence-based analysis of retinal fluid volume dynamics in neovascular age-related macular degeneration and association with vision and atrophy

**DOI:** 10.1038/s41433-024-03399-1

**Published:** 2024-10-15

**Authors:** Siqing Yu, Ian Lloyd Jones, Andreas Maunz, Isabel Bachmeier, Thomas Albrecht, Andreas Ebneter, Martin Gliem, Giovanni Staurenghi, SriniVas R. Sadda, Usha Chakravarthy, Sascha Fauser

**Affiliations:** 1 F. Hoffmann-La Roche Ltd, Basel, Switzerland; 2 University of Bern, Bern, Switzerland; 3 Department of Biomedical and Clinical Science, Luigi Sacco Hospital University of Milan, Milan, Italy; 4 Doheny Image Reading Center, Doheny Eye Institute, Pasadena, CA USA; 5 University of California—Los Angeles, Los Angeles, CA USA; 6 Queens University of Belfast, Belfast, Northern Ireland

**Keywords:** Macular degeneration, Biomarkers

## Abstract

**Background/objectives:**

To characterise morphological changes in neovascular age-related macular degeneration (nAMD) during anti-angiogenic therapy and explore relationships with best-corrected visual acuity (BCVA) and development of macular atrophy (MA).

**Subjects/methods:**

Post-hoc analysis of the phase III HARBOR trial. SD-OCT scans from 1097 treatment-naïve nAMD eyes were analysed. Volumes of intraretinal cystoid fluid (ICF), subretinal hyperreflective material (SHRM), subretinal fluid (SRF), pigment epithelial detachment (PED) and cyst-free retinal volume (CFRV) were measured by deep-learning model. Volumes were analysed by treatment regimen, macular neovascularisation (MNV) subtypes and topographic location. Associations of volumetric features with BCVA and MA development were quantified at month 12/24.

**Results:**

Differences in feature volume changes by treatment regimens and MNV subtypes were observed. Each additional 100 nanolitre unit (AHNU) of residual ICF, SHRM and CFRV at month 1 in the fovea was associated with deficits of 10.3, 7.3 and 12.2 letters at month 12. Baseline AHNUs of ICF, CFRV and PED were associated with increased odds of MA development at month 12 by 10%, 4% and 3%. While that of SRF was associated with a decrease in odds of 5%. Associations at month 24 were similar to those at month 12.

**Conclusion:**

Eyes with different MNV subtypes showed distinct trajectories of feature volume response to treatment. Higher baseline volumes of ICF or PED and lower baseline volume of SRF were associated with higher likelihoods of MA development over 24 months. Residual intraretinal fluid, including ICF and CFRV, along with SHRM were predictors of poor visual outcomes.

## Introduction

Spectral-domain optical coherence tomography (SD-OCT) is currently a pivotal tool in the diagnosis and management of neovascular age-related macular degeneration (nAMD). It provides high-resolution, cross-sectional imaging of the retina, enabling the detection and monitoring of pathological features such as intraretinal cystoid fluid (ICF), subretinal hyperreflective material (SHRM), subretinal fluid (SRF) and pigment epithelial detachment (PED). These features have been shown to be associated with best-corrected visual acuity (BCVA) outcome during anti-angiogenic treatment [1]. ICF and subretinal tissue additionally showed association with anatomical outcome in terms of macular atrophy (MA) development [2].

Typically, these features are manually graded on SD-OCT images. Recent advances in automated image analysis using deep learning (DL) have made it possible to explore the quantitative dynamics of volumes of these features as well as their relationship to BCVA [3]. Initial investigations using DL have been published, including ones that used HARBOR SD-OCT images, which reported that ICF and SRF volumes diminished rapidly under treatment and that BCVA increased accordingly [4, 5]. SHRM segmentation has also been reported recently, but the analysis was performed in small datasets [6, 7]. Furthermore, information regarding the quantitative relationship between these feature volumes and MA remains limited.

The consensus classification of nAMD proposed by the CONAN group recommends the utilisation of SD-OCT images to identify macular neovascularisation (MNV) subtypes [8]. In contrast to fluorescein angiography (FA) where the pattern of leakage is used to assign eyes as exhibiting classic, occult, or mixed MNV, the CONAN classification utilises multimodal imaging to subdivide MNV into type 1, type 2 and type 3. The latter subtype also is known as retinal angiomatous proliferation (RAP). RAP lesions account for some 20% of MNV, present with severe intraretinal exudation and exhibit retinal tissue loss after fluid resolution [9]. This type of response to treatment acts as a confounder when establishing relationships to functional outcome as retinal tissue loss will limit visual recovery. To date, a systematic examination of the dynamics of resolution and persistence of the different components of the exudative lesion by MNV subtypes have not been undertaken.

To address gaps in knowledge, we aimed to provide a comprehensive volumetric analysis of the important SD-OCT features of ICF, SHRM, SRF and PED using the well-phenotyped HARBOR dataset. We also computed the ‘cyst-free retinal volume’ (CFRV) to examine the consequences of diffuse retinal oedema on retinal thinning after treatment initiation. The dynamic changes in volume in the aforementioned features were analysed with respect to MNV subtype, treatment regimen and topographic location. We also assessed the impact of baseline and residual volumes of these features during treatment on BCVA outcome and on the development of MA.

## Methods

The phase III HARBOR trial randomly assigned treatment-naive nAMD patients to four treatment groups: ranibizumab 0.5 mg or 2.0 mg, given monthly or pro re nata (PRN) following a 3-month loading phase. Patients were followed up for 24 months. 14,349 SD-OCT volume scans from 1095 SD-OCT series were obtained using the Cirrus HD-OCT III instrument (Carl Zeiss Meditec, Inc., Dublin, CA), featuring 512 × 128 × 1024 voxels with dimensions 11.7 × 47.2 × 2.0 µm^3^, covering a total volume of 6 × 6 × 2 mm^3^ centred on the fovea. The HARBOR trial design and outcomes have been published in detail [10, 11]. Patients provided written informed consent for their data to be used in future medical research and analyses.

### DL-based SD-OCT segmentation model

To generate ground truth for developing the DL-based model for SD-OCT segmentation, trained graders at the Liverpool Ophthalmology Reading Centre made annotations of ICF, SHRM, SRF, PED and boundaries of neurosensory retina, i.e. internal limiting membrane (ILM), retinal pigment epithelial (RPE) and Bruch’s membrane (BM). Detailed SD-OCT annotation guidance is provided in the Supplementary Method. After the quality assurance processes, 1007 B-scans from a sparse selection of 19 B-scans per volume across 53 volumes with annotations were converted to label maps and used for model development.

The annotated scans were divided into training and holdout/validation sets. To ensure that each feature was represented with approximately equal ratios in both sets, a fraction of 10% of the annotated scans was randomly designated to the holdout set under ICF, SHRM, SRF and PED volume stratifications. All scans of a given patient were allocated to either set in order to avoid overfitting.

The U-Net, a convolutional neural network for biomedical image segmentation [12], was trained to recognize feature classes ICF, SHRM, SRF, PED and neurosensory retina (inner boundary RPE to ILM) on the pixel level (Supplementary Fig. 1 shows semantic segmentation). ICF, SHRM, SRF objects discovered outside ILM and RPE boundaries were removed. For PED, detected objects were constrained between RPE and BM boundaries. The segmentations of all feature classes on B-scans were then reassembled into volumes where each voxel was labelled as belonging to one class.

We introduced a new feature, CFRV, defined as the neurosensory retinal volume minus the volumes of ICF, SHRM and SRF, in order to explore its value with respect to the severity of diffuse retinal oedema at baseline that is not segmented as ICF, as well as the subsequent extent of retinal thinning after fluid resolution.

### Grading of MNV subtypes and MA development

Post hoc image grading was performed to analyse MNV subtypes and MA development based on the most recent consensus definitions of MNV subtypes and MA [13].

The SACCO Reading Center had classified study eyes using criteria consistent with the CONAN consensus classification of nAMD [8]. This reclassification allowed 819 gradable study eyes to be assigned to type 1 (311 eyes), type 2 (303 eyes), type 3 (167 eyes) and mixed (38 eyes) groups. We excluded the mixed group in the fluid dynamics analysis.

The Doheny Image Reading Center had undertaken an assessment for the presence of MA at each visit according to a pre-specified grading protocol [13]. Definite MA was considered in this analysis if three criteria recommended by the classification of macular atrophy (CAM) group were fulfilled: (1) increased signal transmission through the choroid, (2) attenuation of the RPE band, and (3) collapse or thinning of the outer retinal layers [14]. The MA grading allowed 779 gradable study eyes to be categorized into MA present at baseline (119 eyes), MA developed during follow-up (184 eyes), and No MA developed during follow-up (476 eyes).

In the remaining eyes, MNV classification or MA grading was not possible due to either the absence of SD-OCT, FA, or fundus photography (FP) images, or because the available images were of inadequate quality for classification purposes.

### Statistical analysis

Segmentation performance for ICF, SHRM, SRF and PED on the holdout set was assessed against the corresponding reference annotations and measured via a variety of performance metrics: Sørensen–Dice coefficient, positive predictive value, sensitivity and specificity.

Mean volumes of ICF, SHRM, SRF, PED and CFRV over 24 months were analysed in regards to treatment regimen, MNV subtypes and topographic location. For each patient, the within-patient feature volume ‘fluctuation’, defined as the standard deviation (SD) of ICF, SHRM, SRF, PED volume and CFRV from all available visits between month 3 and 24 was calculated. The Kruskal–Wallis test and Dunn pairwise test were used to identify significant differences in fluid volume and fluctuation between subtypes. In addition, mean CFRV over 24 months were analysed in regards to MA development category.

Multilinear regression was used to assess associations of BCVA outcome at month 12 and 24 with volumes of ICF, SHRM, SRF, PED and CFRV at major time points. Logistic regression models were applied to find associations of MA development at month 12 and 24 and volumes of ICF, SHRM, SRF, PED and CFRV at major time points.

All analyses were performed using the R statistical software. *P* values ≤ 0.05 were considered significant.

## Results

Compared to human annotation, our model yielded a mean Dice coefficient of 0.70, 0.69, 0.67 and 0.73 for ICF, SHRM, SRF and PED, respectively. Supplementary Fig. 2 shows the performance of our model on the holdout set.

**Fig. 1:**
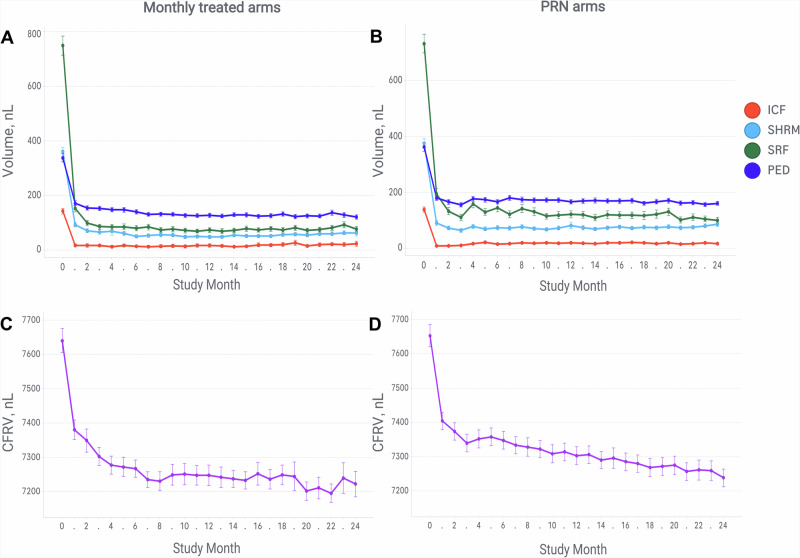
Mean volumetric features over 24 months in the monthly and pro re nata arms. Mean volumes of intraretinal cystoid fluid (ICF), subretinal hyperreflective material (SHRM), subretinal fluid (SRF) and pigment epithelial detachment (PED) over 24 months in (**A**) monthly-treated arms and **B** pro re nata (PRN) arms. Cyst-free retinal volume (CFRV) in (**C**) monthly-treated arms and **D** PRN arms. Error bars indicate standard error.

All feature volumes decreased rapidly between baseline and month 1, further decreasing until month 3 for both treatment regimens. Minor increases in all feature volumes were observed after the loading phase in the PRN arms but not in the monthly-treated arms. CFRV showed a continual decrease during the entire follow-up (Fig. 1). Residual volumes of all features tended to be higher in PRN arms than in monthly arms. At month 12, the differences were significant for SHRM, SRF and PED. (Supplementary Table 1).

**Fig. 2:**
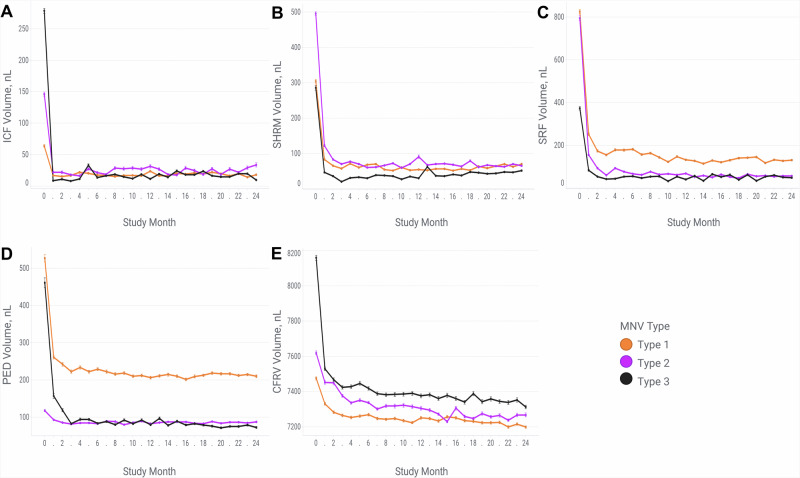
Mean volumetric features over 24 months in the different macular neovascularisation subtypes. Mean volumes of (**A**) intraretinal cystoid fluid (ICF), **B** subretinal hyperreflective material (SHRM), **C** subretinal fluid (SRF), **D** pigment epithelial detachment (PED) and **E** cyst-free retinal volume (CFRV) over 24 months in eyes with different macular neovascularisation (MNV) subtypes. Error bars indicate standard error.

MNV type influenced fluid and SHRM volumes (Fig. 2 and Supplementary Table 2 show volumes at major time points). At baseline, Type 1 MNVs were characterized as having significantly lower ICF volume and CFRV and higher PED volume; Type 2 MNVs were characterized by significantly higher SHRM and lower PED volume; Type 3 MNVs were distinguished by significantly higher ICF volume, CFRV and lower SRF volume. After the loading phases, the differences in ICF volume and CFRV lost significance. However, eyes with type 1 MNVs had significantly more residual SRF and PED volume until the end of the follow-up, compared to the eyes with type 2 and 3 MNVs. Regarding the volume fluctuation during the maintenance phase, we observed that type 2 MNVs had significantly larger SHRM volume fluctuation compared to type 3 MNVs, while type 1 MNVs showed significantly greater fluctuation in SRF and PED volume compared to other MNV types (Supplementary Table 3).

ICF, SHRM, SRF and PED exhibited heterogeneous distributions within the Early Treatment Diabetic Retinopathy Study (ETDRS) grid (Supplementary Table 4 and Supplementary Fig. 4). At baseline, the majority of SHRM, ICF and PED were located within the central 1 mm circle. In contrast, SRF was distributed across most sectors of the ETDRS grid, indicating a more widespread distribution.

Table 1 shows the mean effect on month 12 BCVA of each additional 100 nL nanolitre unit (AHNU) of feature volume. ICF, CFRV and SHRM showed constant negative associations with worse BCVA at all major time points. The volumes in the foveal central subfield (FCS) and the residual volumes starting from month 1 had the strongest impact on BCVA. On the contrary, SRF and PED volume showed treatment-phase-dependent effects on BCVA. At baseline, SRF and PED volume showed minor associations with BCVA, while from month 6 on, significantly positive associations between residual SRF or PED volume in the FCS and BCVA outcome were observed. The feature volumes showed similar associations with month 24 BCVA (Supplementary Table 5).

**Table 1:** Multilinear regression model estimates associations of every additional 100 nL of feature volumes at main time points on BCVA at month 12.

Feature	ETDRS grid	Mean coefficient (SE)				
		Baseline (*n* = 986)	Month 1 (*n* = 979)	Month 3 (*n* = 976)	Month 6 (*n* = 968)	Month 9 (*n* = 964)
ICF	1 mm	−5.8 (−7.7, −3.9)***	−10.3 (−22.1, −0.1)*	−11.2 (−21.3, 3.1)*	−10.2 (−17.7, −2.7)**	−10.2 (−18.4, −2)*
	3 mm	−1.5 (−2.1, −0.7)***	−1.3 (−4.5, 1.7)	−0.9 (−3.9, 2.8)	−2.9 (−5, −0.8)**	−1.7 (−3.6, 0.3)
	6 mm	−1.3 (−1.9, −0.6)***	−0.8 (−3.8, 1.2)	−1.1 (−3.5, 1.1)	−2 (−3.6, −0.5)*	−1 (−2.2, 0.2)
SHRM	1 mm	−3.9 (−5.9, −2)***	−12.2 (−17.2, −8.1)***	−13.4 (−20.1, −8)***	−17.9 (−23.5, −12.4)***	−14.3 (−20.1, −8.5)***
	3 mm	−1.3 (−1.7, −0.9)***	−3.3 (−4.4, −2.4)***	−3.8 (−5.2, −2.7)***	−3.9 (−4.9, −2.8)***	−4.2 (−5.4, −3)***
	6 mm	−1 (−1.3, −0.7)***	−2.4 (−3.1, −1.8)***	−1.9 (−3.3, −0.9)***	−2.8 (−3.6, −2.1)***	−2.3 (−3, −1.6)***
SRF	1 mm	−3.3 (−6.1, −0.8)**	2.6 (−0.7, 5.9)	4.4 (−1.2, 8.9)	6.4 (1.8, 11)**	5.7 (1.2, 10.3)*
	3 mm	−0.5 (−0.9, 0)**	0 (−0.7, 0.7)	0.2 (−0.9, 1.2)	1.2 (0.2, 2.2)*	1.3 (0.3, 2.3)*
	6 mm	−0.1 (−0.3, 0)	−0.1 (−0.4, 0.2)	−0.1 (−0.6, 0.3)	0.4 (0, 0.8)	0.4 (0, 0.9)
PED	1 mm	−0.4 (−2.5, 1.2)	1.4 (−1.3, 3.8)	3.5 (−0.1, 5.7)*	4.9 (2.1, 7.7)***	5.8 (3, 8.6)***
	3 mm	−0.1 (−0.5, 0.1)	0 (−0.6, 0.6)	0.4 (−0.4, 1)	0.3 (−0.3, 0.9)	0.5 (−0.1, 1.1)
	6 mm	0 (−0.3, 0.1)	0.1 (−0.4, 0.5)	0.2 (−0.3, 0.7)	0.1 (−0.3, 0.6)	0.1 (−0.4, 0.6)
CFRV	1 mm	−5.4 (−7.8, −2.9)***	−7.3 (−10.5, −4.1)***	−7.4 (−11.1, −3.7)***	−6.2 (−9.1, −3.4)***	−6.7 (−10.1, −3.4)***
	3 mm	−0.2 (−0.6, 0.2)	−0.5 (−1.1, 0)*	−0.7 (−1.2, −0.1)*	−0.5 (−1, 0)	−0.5 (−1.1, 0)*
	6 mm	0 (−0.1, 0.2)	0 (−0.2, 0.2)	0 (−0.2, 0.2)	0 (−0.2, 0.2)	0 (−0.2, 0.2)

Data shown in mean ETDRS letters (95% CI).

*BCVA* best-corrected visual acuity, *CFRV* cyst-free retinal volume, *CI* confidence interval, *ETDRS* Early Treatment Diabetic Retinopathy Study, *ICF* intraretinal cystoid fluid, *nL* nanoliter, *PED* pigment epithelial detachment, *SHRM* subretinal hyperreflective material, *SRF* subretinal fluid.

*p < 0.05, **p < 0.01, ***p < 0.001.

Quantitative associations between volumetric features and new MA development were assessed (Table 2). Supplementary Fig. 4 shows how eyes were selected for logistic regression analysis. At baseline, each AHNU of ICF, PED and CFRV was associated with an increased odds ratio (OR) of MA development at month 12 by 10% (OR 1.1; 95%-CI, 1.01–1.2), 4% (OR 1.04; 95%-CI, 1.01–1.08) and 3% (OR 1.03; 95%-CI, 1.0–1.06). For each AHNU of SRF volume at baseline, the odds of MA onset decreased by 5% (OR 0.95; 95%-CI, 0.92–0.98). Residual volumes of ICF, PED and CFRV and at the later time points exhibited no significant associations with MA. No statistically significant association was found between SHRM at any visits on MA development. The association with MA development over 24 months was weaker but comparable to that of the first 12 months (Supplementary Table 6).

**Table 2:** Logistic regression model estimates associations of every additional 100 nL of feature volumes in the central 6 mm (diameter) grid at main time points on MA development at month 12.

Feature	Baseline (*n* = 689)		Month 1 (*n* = 656)		Month 3 (*n* = 614)		Month 6 (*n* = 589)		Month 9 (*n* = 563)	
	OR (95% CI)	*P* value	OR (95% CI)	*P* value	OR (95% CI)	*P* value	OR (95% CI)	*P* value	OR (95% CI)	*P* value
ICF	**1**.**1** (**1**.**01**, **1**.**2**)	**0**.**032**	0.78 (0.41, 1.5)	0.461	1.08 (0.75, 1.54)	0.688	0.72 (0.17, 3.06)	0.659	0.49 (0.02, 14.4)	0.682
SHRM	0.99 (0.93, 1.05)	**0**.795	1.04 (0.91, 1.19)	0.583	1.06 (0.89, 1.27)	0.518	1.13 (0.88, 1.45)	0.335	1.15 (0.82, 1.61)	0.412
SRF	**0**.**95** (**0**.**92**, **0**.**98**)	**0**.**003**	0.95 (0.88, 1.03)	0.222	0.97 (0.82, 1.14)	0.688	0.95 (0.77, 1.17)	0.635	0.31 (0.04, 2.12)	0.231
PED	**1**.**04** (1.**01**, **1**.**08**)	**0**.**004**	0.99 (0.91, 1.08)	0.857	0.93 (0.79, 1.09)	0.363	0.84 (0.63, 1.14)	0.271	0.77 (0.41, 1.46)	0.422
CFRV	**1**.**03** (1, 1.**06**)	**0**.**021**	1.04 (1, 1.08)	0.075	1.03 (0.99, 1.08)	0.188	0.95 (0.87, 1.02)	0.163	0.99 (0.88, 1.12)	0.921

OR values with significant *p* values (under 0.05) are in bold font.

*CFRV* cyst-free retinal volume, *CI* confidence interval, *ICF* intraretinal cystoid fluid, *MA* macular atrophy, *OR* odds ratio, *PED* pigment epithelial detachment, *SHRM* subretinal hyperreflective material, *SRF* subretinal fluid.

**Fig. 3:**
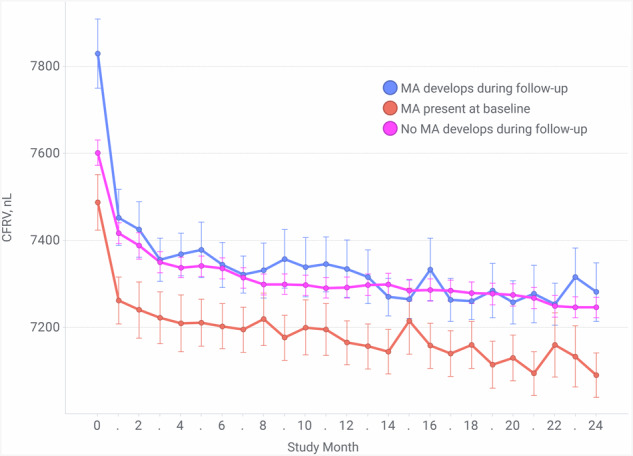
CFRV over 24 months in the eyes grouped by MA development categories. Mean cyst-free retinal volume (CFRV) over 24 months in eyes grouped by different macular atrophy (MA) development. CFRV in the eyes where no MA development were significantly higher than the eyes in the other two groups. Error bars indicate standard error.

Because lower CFRV values may be a sign of retinal thinning due to MA, we grouped eyes by MA presence at baseline and development during follow-up. Baseline CFRV was the highest in the eyes that developed MA during the follow-up, followed by the eyes that never developed MA, and was the lowest in the eyes showing MA already at baseline. In the eyes showing MA at baseline, CFRV was lower across all visits compared to the other groups (Fig. 3).

## Discussion

We used a DL-based model to automatically segment ICF, SHRM, SRF, PED and CFRV volumes using SD-OCT images from the HARBOR dataset. We examined these feature volume distributions and changes during the 24-month anti-angiogenic treatment across several dimensions: MNV type, treatment regimen, dose and topographic location. We demonstrated a large-scale comprehensive fluid volumetric analysis of a wide range of features, with their impact on BCVA. Additionally, we presented the following novel results: First, we demonstrated the differences in the trajectories of feature volume response to treatment by MNV subtype. Second, we described relationships between feature volumes and MA development. Third, we derived a new feature the CFRV, which permitted us to account for diffuse oedema at the beginning of the treatment, and for retinal thinning after the fluid resolution.

Previous studies have shown a negative impact of ICF on BCVA and an association with MA development [2, 4]. We not only confirmed these associations but also provided the quantitative effects based on the severity of this parameter. Most ICF resolved rapidly in the parafoveal regions by month 1 after treatment initiation. However, residual ICF in the FCS exhibited a strong negative association with BCVA. Reasons may include suboptimal inhibition of leakage from neovascular complexes resulting in persistence of loculated fluid or degeneration of the neurosensory retina with fluid from a non-neovascular origin.

In contrast to ICF segmentation, evaluation of diffuse oedema has been an unresolved problem. To date, DL-based segmentation has only segmented clearly distinguished cystoid spaces as intraretinal fluid [3]. We therefore explored a novel parameter, the CFRV to test if it could be used as a potential proxy for diffuse macular oedema. We observed that CFRV, like ICF, declined rapidly after the first injection and demonstrated similar negative associations with BCVA. In contrast with other features of exudation such as ICF, SRF and SHRM, for which a steady state was reached by month 3, CFRV after the initial rapid decrease subsequently continued to reduce during the entire follow-up, especially in eyes with MA at baseline and those that developed MA during follow-up. We contend that CFRV could be considered a proxy for the severity of diffuse oedema at initiation of treatment, and subsequently also as a marker for retinal thinning after fluid resolution.

Presence or larger size of SHRM has been reported as negative morphologic biomarkers in nAMD [15]. The current study reinforced this message by demonstrating the detrimental effect of SHRM volume on BCVA, especially when located in the FCS. SHRM in treatment-naive eyes is composed of resolvable and unresolvable elements. Following treatment, as the fluid elements of SHRM resolve, SHRM changes from fluid-dominated to fibrosis-dominant (angiofibrotic switch) [16]. Previously, we have observed that baseline SHRM resolved within 3 months of anti-angiogenic treatment in around one-third of the treatment-naive eyes, and meanwhile the boundaries of SHRM changed rapidly from undefined to well-defined within 1 month in the other two-thirds of the eyes where SHRM stayed persistent [17]. In the current study, we demonstrated that SHRM volume decreases rapidly after the first injection, further decline is shallow until month 3, without much change thereafter, which corresponds to our previous findings of SHRM resolution and boundary change. Our data on the dynamics of SHRM resolution or persistence while supporting its role as an important biomarker in nAMD trials, also provides quantitative data to define clinical trial endpoints.

MA development has been considered to develop in advanced stages of subretinal fibrosis [18]. A CATT subset analysis showed that the presence of baseline SHRM was associated with increased odds of MA development [19]. Casalino et al. showed thicker SHRM at baseline was a risk factor of MA development [20]. However, our volumetric analysis did not show significant association between baseline or residual SHRM volume at all three times with new MA development. Notably, the results from these studies are not completely comparable, as inconsistent definitions of MA, fibrosis or SHRM, and different methodology to assess these features were applied. Nevertheless, our results highlight the complex association between SHRM or fibrosis with MA development. Previously, we observed hyperreflective material boundary remodelling (HRM-BR) in about half of the eyes with persistent SHRM, and these eyes were associated with better BCVA and less MA [17]. Further volumetric analysis considering more perspectives of SHRM, such as HRM-BR, is needed to better understand the association between SHRM and MA development.

Unlike intraretinal fluid, SRF has been shown to be less detrimental to visual outcome. In the FLUID trial, similar BCVA outcomes were achieved between arms tolerating minimal residual SRF or resolving all SRF [21]. However, the post hoc volumetric analysis of the FLUID trial showed SRF volume had a minor but significant negative effect on BCVA [22]. Therefore the protective value of SRF in nAMD regarding BCVA remains uncertain. Our volumetric analysis showed treatment-phase-dependent effects of SRF volume on BCVA: baseline SRF volume in the FCS had a negative effect on BCVA, whereas residual SRF from month 6 on showed significant negative association with BCVA.

Baseline and residual SRF were reported associated with a lower risk for MA development and progression [2, 23]. Similarly, we observed that SRF volume at all three visits was associated with decreased odds of MA development, although only significantly at baseline. Of note, the HARBOR data had been specifically analysed for both prevalent and incident MA using the CAM criteria based on SD-OCT. Therefore, it reduced the probability of misclassifications that may occur with FP and FA. One hypothesis of the protective effect of SRF is that the presence of small amounts of residual SRF indicates a persistent but controlled MNV lesion below an intact RPE layer, and therefore portends a more favourable anatomic outcome with preservation of function in the nAMD patient [24]. Nonetheless, to better understand the relationships between SRF volume and BCVA or MA development, further analysis is still necessary.

Baseline PED was not associated with BCVA outcome, but was significantly associated with higher odds of MA development. On the contrary, residual PED volume in the FCS from month 6 on was significantly associated with better BCVA outcome, but was not associated with MA development. We assume the MA associations with baseline PED were due to the presence of very large vascularized PEDs that were reported to be more likely to undergo collapse following anti-VEGF treatment [25]. While during the maintenance phase, residual PED in the FCS indicates both an intact RPE layer and the presence of a vascular net external to the RPE, which could explain the positive association with visual outcome [26].

In this work, we characterized the volumetric features in different MNV subtypes in a large dataset, which has not been reported before. At baseline, ICF and CFRV, both representing intraretinal exudation, were significantly prominent in type 3 MNVs. SHRM volumes were significantly higher and fluctuated more in type 2 MNVs, in accord with the view that SHRM represents the SD-OCT-correlate of the subretinal portion of the neovascular complexes. Significantly larger PED volumes were seen in type 1 and 3 MNVs and are consistent with current knowledge on the location of the neovascular complexes in these nAMD subtypes. On completion of the loading phase, type 3 MNVs did not exhibit higher ICF, PED volume and CFRV, and notably experienced lesser fluctuations of SHRM, SRF and PED volumes, indicating greater resolution of intraretinal and sub-RPE exudation in this subtype, which corroborates earlier reports of better treatment responses in type 3 MNV [9].

The quantitative association between volumetric features and MA development that we describe may help better understand MA development in different MNV types. Our results and those of previous studies [9], indicate that type 3 MNV manifests with higher ICF and PED volumes and lower SRF volume at baseline, all of which are associated with increased odds of MA development. Contrarily, type 1 MNV manifests with higher SRF volume and lower ICF volume at baseline, associated with decreased odds of MA development.

Our results provide straightforward information for understanding MNV subtypes proposed by the CONAN group, which previously required extensive clinical experience. It also reinforces again the reliability of SD-OCT images as a standalone tool in MNV classification [27], reducing the need for invasive FA imaging.

Notably, we also observed the benefits of the monthly regimen over the PRN in reducing residual feature volumes, which was not revealed in the primary analysis of HARBOR, where only central foveal thickness and BCVA were compared [10, 11]. As the FLUID study showed, tolerating some residual SRF achieved non-inferior visual outcomes with less frequent treatment, while such tolerated residual SRF had a minor but significant negative effect on BCVA [21, 22]. Though the study designs were different, the findings from the FLUID trial and our study suggested eyes treated with less frequent injections, such as PRN, are more likely to suffer from higher amounts of residual exudation, which potentially has a negative impact on vision.

This study is limited in its retrospective nature. The performance of our model in detecting and segmenting ICF, SHRM, SRF and PED, was comparable to model performance in previous studies [28]. However, the main limitation of our model includes the absence of multiple annotations and an independent validation set. Though the OCT images obtained in the HARBOR trial were focused on the foveal centre, there is a possibility of erroneous centration introducing bias into the topography analysis.

Although the volumetric features were statistically significantly associated with BCVA and MA, the coefficients and ORs are not large. This indicates the likelihood of presence of other biomarkers which may be contributing to the BCVA outcome, such as photoreceptor loss at the fovea, hyperreflective foci, fibrosis formation, etc. Further investigation considering these additional features would provide a deeper understanding of visual outcome and MA development.

In summary, eyes with different MNV subtypes presented distinct trajectories of feature volume response to treatment. During the 24-month observational period, higher baseline and residual intraretinal fluid, including ICF and CFRV, together with SHRM volumes were linked to worse visual outcome. Higher baseline volumes of intraretinal fluid or PED and lower baseline volumes of SRF were associated with a greater likelihood of MA development.

## Summary

### What was known before

- ICF, SRF and PED are associated with visual outcome in the eyes with nAMD, and artificial intelligence-based approaches can segment these features volumetrically.
- MNV subtypes are discernible on OCT scans; they are characterized by the presence and amount of ICF, SRF, PED and SHRM.
- Absence of SRF, presence of ICF and presence of type 3 MNV are associated with macular atrophy development.

### What this study adds

- In this comprehensive volumetric analysis of a large dataset, we are the first to report the volumetric fluid dynamics including ICF, SRF, PED and SHRM in eyes grouped by different MNV subtypes. Our results provide straightforward insights to increase the understanding of MNV subtypes.
- We also provide the volumetric fluid-quantified odds ratio of MA development, and introduced the novel feature ‘cyst-free retina volume’ for calculating retinal thinning following fluid resolution.

## Supplementary information

Supplemental Method. SD-OCT annotation guidance followed by the Liverpool Ophthalmology Reading Centre.

Supplemental Table 1. Mean Residual Volumes of ICF, SHRM, SRF, PED and CFRV in nL (±SE) at Month 12 and 24 in the Eyes Treated Monthly or PRN.

Supplemental Table 2. Mean Volumes of ICF, SHRM, SRF, PED and CFRV at Main Time Points in All Eyes and per MNV Subtype.

Supplemental Table 3. Median (IQR) SD of ICF, SHRM, SRF, PED and CFRV during the maintenance phase (Month 3–24) per MNV Subtype.

Supplemental Table 4. Distribution of ICF, SHRM, SRF and PED in the ETDRS Grid for Monthly Treated Eyes at Main Time Points.

Supplemental Table 5. Multilinear Regression Model Estimates Associations of Every Additional 100 Nanoliter of Feature Volumes Distributed in ETDRS Grid at Main Time Points on BCVA at Month 24.

Supplemental Table 6. Logistic Regression Model Estimates Associations of Every Additional 100 nL of Feature Volumes in the Central 6 mm (diameter) Grid at Main Time Points on MA Development at Month

Supplemental Figure Legends

Supplemental Figure 1. Illustration of The Process to Generate Volumetric Features From SD-OCT Volume Scans.

Supplemental Figure 2. Feature Segmentation Performance.

Supplemental Figure 3. Distribution of Volumetric Features in the Early Treatment Diabetic Retinopathy Study Grid.

Supplemental Figure 4. Feature funneling: selection process for data used to develop the logistic regression models estimates associations of feature volumes on MA development at Month 12.
