# Correction: Artificial intelligence-based analysis of retinal fluid volume dynamics in neovascular age-related macular degeneration and association with vision and atrophy

**DOI:** 10.1038/s41433-025-04232-z

**Published:** 2026-01-21

**Authors:** Siqing Yu, Ian Lloyd Jones, Andreas Maunz, Isabel Bachmeier, Thomas Albrecht, Andreas Ebneter, Martin Gliem, Giovanni Staurenghi, SriniVas R. Sadda, Usha Chakravarthy, Sascha Fauser

**Affiliations:** 1 F. Hoffmann-La Roche Ltd, Basel, Switzerland; 2 University of Bern, Bern, Switzerland; 3 Department of Biomedical and Clinical Science, Luigi Sacco Hospital University of Milan, Milan, Italy; 4 Doheny Image Reading Center, Doheny Eye Institute, Pasadena, CA USA; 5 University of California—Los Angeles, Los Angeles, CA USA; 6 Queens University of Belfast, Belfast, Northern Ireland

Correction to:* Eye* 10.1038/s41433-024-03399-1, published online 15 October 2024

In this article the author name “Usha Chakravarthy” was incorrectly written as “Usha Chakravarty”.

The original article has been corrected.

