# Supplementary material for: Artificial intelligence-based analysis of retinal fluid volume dynamics in neovascular age-related macular degeneration and association with vision and atrophy
Source: Eye (Lond). 2024 Oct 15;39(1):154–61. doi: 10.1038/s41433-024-03399-1 (PMC11732971; doi:10.1038/s41433-024-03399-1)
Supplement: Supplementary file 1 — Supplemental Method. SD-OCT annotation guidance followed by the Liverpool Ophthalmology Reading Centre. [file 41433_2024_3399_MOESM1_ESM.docx]

**Supplemental method. SD-OCT annotation guidance followed by the Liverpool Ophthalmology Reading Centre**

Two trained annotators made the following annotations under supervision of a senior ophthalmologist on 1007 B-scans from a sparse selection of 19 B-scans per volume across 53 volumes. A custom-designed software (developed by Department of Eye and Vision Science, University of Liverpool) was used to facilitate the annotation.

1. Segmentation of the following retinal layers was undertaken on all selected B-scans. Specific color was allocated to each of the layers, and it was ensured that lines do not cross over.

- Internal Limiting Membrane (ILM)
- Outer border of Outer Plexiform Layer (OPL)
- External Limiting Membrane (ELM)
- Ellipsoid zone (EZ)
- Outer border of retinal pigment epithelium (RPE)
- Bruch’s membrane

2. intraretinal cystoid fluid (ICF)

ICF is defined as circular hyporeflective spaces that are individually equal to or more than 50μm in size. These are to be drawn separately with no overlap between individual cystic spaces. If the ICF spaces are close together, then zoom into the image to allow a better view of difficult areas.

3. Subretinal Hyper-reflective Material (SHRM)

SHRM is defined as areas of varying hyper-reflectivity within its boundaries and is distinguishable from surrounding neural components.

4. Subretinal fluid (SRF)

SRF is defined as areas of non-reflectivity or moderate reflectivity between the neurosensory retina and RPE. These annotations are to be drawn as separate areas and with no overlap of lines.

5. Pigment epithelium detachment (PED)

PED is defined as elevation of the RPE. These annotations are to be drawn as separate areas and with no overlap of lines. Multiple PEDs would be drawn as separate annotations.
