# Supplementary material for: Artificial intelligence-based analysis of retinal fluid volume dynamics in neovascular age-related macular degeneration and association with vision and atrophy
Source: Eye (Lond). 2024 Oct 15;39(1):154–61. doi: 10.1038/s41433-024-03399-1 (PMC11732971; doi:10.1038/s41433-024-03399-1)
Supplement: Supplementary file 2 — Supplemental Table 1. Mean Residual Volumes of ICF, SHRM, SRF, PED and CFRV in nL (±SE) at Month 12 and 24 in the Eyes Treated Monthly or PRN. [file 41433_2024_3399_MOESM2_ESM.docx]

**Supplemental Table 1. Mean Residual Volumes of ICF, SHRM, SRF, PED and CFRV in nL (± SE) at Main Time Points in the Eyes Treated Monthly or PRN.**

|  |  | Month 12 | | | Month 24 | | |
| --- | --- | --- | --- | --- | --- | --- | --- |
| MNV type | Feature | (Monthly arms cohort size) | (PRN arms cohort size) | p-value | (Monthly arms cohort size) | (PRN arms cohort size) | p-value |
| All Types |  | (n = 495) | (n = 493) |  | (n = 461) | (n = 463) |  |
|  | ICF | 17 ± 6 | 22 ± 3 | 0.52 | 24 ± 9 | 19 ± 3 | 0.57 |
|  | SHRM | **49 ± 6** | **83 ± 11** | **0.01** | 63 ± 10 | 87 ± 8 | 0.07 |
|  | SRF | **74 ± 8** | **123 ± 12** | **0.00** | 78 ± 9 | 102 ± 10 | 0.08 |
|  | PED | **128 ± 10** | **167 ± 11** | **0.01** | **122 ± 10** | **162 ± 11** | **0.01** |
|  | CFRV | 7248 ± 30 | 7304 ± 26 | 0.16 | 7223 ± 37 | 7239 ± 26 | 0.72 |
| Type 1 |  | (n = 133) | (n = 148) |  | (n = 125) | (n = 136) |  |
|  | ICF | 16 ± 8 | 26 ± 7 | 0.34 | 15 ± 4 | 20 ± 5 | 0.43 |
|  | SHRM | 45 ± 10 | 62 ± 11 | 0.24 | 53 ± 10 | 85 ± 16 | 0.08 |
|  | SRF | **107 ± 22** | **184 ± 27** | **0.03** | 118 ± 23 | 142 ± 24 | 0.46 |
|  | PED | 185 ± 24 | 251 ± 27 | 0.06 | 191 ± 25 | 230 ± 22 | 0.25 |
|  | CFRV | 7190 ± 44 | 7252 ± 49 | 0.35 | 7142 ± 44 | 7213 ± 52 | 0.30 |
| Type 2 |  | (n = 137) | (n = 137) |  | (n = 130) | (n = 127) |  |
|  | ICF | 33 ± 21 | 24 ± 5 | 0.67 | 45 ± 30 | 16 ± 4 | 0.35 |
|  | SHRM | 56 ± 12 | 121 ± 37 | 0.10 | 56 ± 8 | 75 ± 12 | 0.18 |
|  | SRF | **47 ± 12** | **86 ± 14** | **0.03** | 55 ± 14 | 72 ± 13 | 0.37 |
|  | PED | **72 ± 7** | **100 ± 11** | **0.04** | **71 ± 8** | **104 ± 11** | **0.02** |
|  | CFRV | 7307 ± 78 | 7314 ± 50 | 0.94 | 7273 ± 84 | 7258 ± 46 | 0.87 |
| Type 3 |  | (n = 77) | (n = 75) |  | (n = 70) | (n = 72) |  |
|  | ICF | **3 ± 1** | **19 ± 7** | **0.03** | 8 ± 5 | 11 ± 2 | 0.64 |
|  | SHRM | 27 ± 7 | 35 ± 7 | 0.45 | 39 ± 9 | 59 ± 15 | 0.23 |
|  | SRF | 35 ± 8 | 36 ± 14 | 0.92 | 52 ± 18 | 41 ± 8 | 0.58 |
|  | PED | 96 ± 24 | 84 ± 13 | 0.65 | 88 ± 27 | 81 ± 11 | 0.81 |
|  | CFRV | 7328 ± 66 | 7378 ± 63 | 0.59 | 7437 ± 130 | 7312 ± 62 | 0.39 |

Abbreviations: CFRV, cyst-free retinal volume; ICF, intraretinal cystoid fluid; MNV, macular neovascularization; nL, nanoliter; PED, pigment epithelial detachment; PRN, pro re nata; SE, standard error; SHRM, subretinal hyperreflective material; SRF, subretinal fluid.

Two-sided Student's t-Test was used.
