# Supplementary material for: Artificial intelligence-based analysis of retinal fluid volume dynamics in neovascular age-related macular degeneration and association with vision and atrophy
Source: Eye (Lond). 2024 Oct 15;39(1):154–61. doi: 10.1038/s41433-024-03399-1 (PMC11732971; doi:10.1038/s41433-024-03399-1)
Supplement: Supplementary file 3 — Supplemental Table 2. Mean Volumes of ICF, SHRM, SRF, PED and CFRV at Main Time Points in All Eyes and per MNV Subtype. [file 41433_2024_3399_MOESM3_ESM.docx]

**Supplemental Table 2. Mean Volumes of ICF, SHRM, SRF, PED and CFRV at Main Time Points in All Eyes and per MNV Subtype.**

| Feature | MNV Subtype | Mean volume (SE), nL | | | | | | | | | |
| --- | --- | --- | --- | --- | --- | --- | --- | --- | --- | --- | --- |
|  |  | Baseline | | Month 1 | | Month 3 | | Month 12 | | Month 24 | |
|  |  | Mean (SE) | N | Mean (SE) | N | Mean (SE) | N | Mean (SE) | N | Mean (SE) | N |
| ICF | All Types | 148 (11) | 385 | 22 (6) | 376 | 20 (6) | 370 | 20 (9) | 347 | 25 (12) | 325 |
|  | MNV 1 | 70 (11)^2, 3^ | 147 | 21 (8) | 146 | 22 (10) | 144 | 16 (8) | 133 | 15 (4) | 125 |
|  | MNV 2 | 163 (22)^1, 3^ | 151 | 30 (14) | 146 | 21 (10) | 144 | 33 (21) | 137 | 45 (30) | 130 |
|  | MNV 3 | 252 (22)^1, 2^ | 87 | 10 (3) | 84 | 15 (5) | 82 | 3 (1) | 77 | 8 (5) | 70 |
| SHRM | All Types | 382 (20) | 385 | 99 (10) | 376 | 68 (12) | 370 | 46 (6) | 347 | 51 (5) | 325 |
|  | MNV 1 | 285 (29)^2^ | 147 | 84 (14)^2, 3^ | 146 | 84 (27)^3^ | 144 | 45 (10) | 133 | 53 (10) | 125 |
|  | MNV 2 | 535 (36)^1, 3^ | 151 | 145 (20)^1, 3^ | 146 | 75 (12)^3^ | 144 | 56 (12)^3^ | 137 | 56 (8) | 130 |
|  | MNV 3 | 280 (34)^2^ | 87 | 47 (13)^1, 2^ | 84 | 26 (5)^1, 2^ | 82 | 27 (7)^2^ | 77 | 39 (9) | 70 |
| SRF | All Types | 770 (42) | 385 | 152 (14) | 376 | 88 (11) | 370 | 67 (10) | 347 | 79 (11) | 325 |
|  | MNV 1 | 892 (64)^3^ | 147 | 182 (27)^3^ | 146 | 134 (25)^3^ | 144 | 107 (22) | 133 | 118 (23)^3^ | 125 |
|  | MNV 2 | 874 (80)^3^ | 151 | 162 (22)^3^ | 146 | 57 (8) | 144 | 47 (12) | 137 | 55 (14) | 130 |
|  | MNV 3 | 383 (48)^1, 2^ | 87 | 81 (16)^1, 2^ | 84 | 63 (22)^1^ | 82 | 35 (8) | 77 | 52 (18)^1^ | 70 |
| PED | All Types | 343 (26) | 385 | 176 (13) | 376 | 147 (13) | 370 | 121 (11) | 347 | 121 (12) | 325 |
|  | MNV 1 | 504 (48)^2, 3^ | 147 | 264 (26)^2, 3^ | 146 | 223 (25)^2, 3^ | 144 | 185 (24)^2, 3^ | 133 | 191 (25)^2, 3^ | 125 |
|  | MNV 2 | 139 (19)^1, 3^ | 151 | 103 (11)^1, 3^ | 146 | 83 (8)^1^ | 144 | 72 (7)^1^ | 137 | 71 (8)^1^ | 130 |
|  | MNV 3 | 425 (64)^1, 2^ | 87 | 151 (27)^1, 2^ | 84 | 125 (31)^1^ | 82 | 96 (24)^1^ | 77 | 88 (27)^1^ | 70 |
| CFRV | All Types | 7656 (42) | 385 | 7384 (35) | 376 | 7310 (31) | 370 | 7267 (38) | 347 | 7258 (47) | 325 |
|  | MNV 1 | 7399 (50)^2, 3^ | 147 | 7252 (43)^2, 3^ | 146 | 7197 (46)^3^ | 144 | 7190 (44) | 133 | 7142 (44) | 125 |
|  | MNV 2 | 7637 (72)^1, 3^ | 151 | 7443 (68)^1^ | 146 | 7354 (52) | 144 | 7307 (78) | 137 | 7273 (84) | 130 |
|  | MNV 3 | 8126 (86)^1, 2^ | 87 | 7510 (65)^1^ | 84 | 7429 (63)^1^ | 82 | 7328 (66) | 77 | 7437 (130) | 70 |

Abbreviations: CFRV, cyst-free retinal volume; ICF, intraretinal cystoid fluid; MNV, macular neovascularization; PED, pigment epithelial detachment; nL, nanoliter; SE, standard error; SHRM, subretinal hyperreflective material; SRF, subretinal fluid.

Kruskal-Wallis test and Dunn pairwise test were used. ^1^ Values are significantly different from type 1 MNV; ^2^ Values are significantly different from type 2 MNV; ^3^ Values are significantly different from type 3 MNV.
