# Supplementary material for: Artificial intelligence-based analysis of retinal fluid volume dynamics in neovascular age-related macular degeneration and association with vision and atrophy
Source: Eye (Lond). 2024 Oct 15;39(1):154–61. doi: 10.1038/s41433-024-03399-1 (PMC11732971; doi:10.1038/s41433-024-03399-1)
Supplement: Supplementary file 4 — Supplemental Table 3. Median (IQR) SD of ICF, SHRM, SRF, PED and CFRV during the maintenance phase (Month 3–24) per MNV Subtype. [file 41433_2024_3399_MOESM4_ESM.docx]

**Supplemental Table 3. Median (IQR) SD of ICF, SHRM, SRF, PED and CFRV during the maintenance phase (Month 3–24) per MNV Subtype.**

|  | Median SD (IQR) | | |
| --- | --- | --- | --- |
|  | Type 1 (n = 147) | Type 2 (n = 149) | Type 3 (n = 84) |
| ICF | 2.6 (0.9, 11.7) | 2.7 (0.9, 8.9) | 2.9 (0.7, 10.7) |
| SHRM | 21.7 (9.7, 45.1) | 27.6 (11.0, 63.7)^3^ | 16.9 (6.6, 41.7)^2^ |
| SRF | 29.3 (13.9, 108.6)^3^ | 24.8 (12.1, 51.8) | 17.0 (6.4, 46.5)^1^ |
| PED | 26.1 (13.8, 50.9)^2, 3^ | 13.6 (6.4, 26.8)^1^ | 12.5 (5.9, 27.8)^1^ |
| CFRV | 93.5 (62.5, 143.7)^2^ | 109.2 (81.7, 158.2)^1^ | 96.7 (66.2, 153.2) |

Abbreviations: CFRV, cyst-free retinal volume; ICF, intraretinal cystoid fluid; IQR, interquartile range; MNV, macular neovascularization; PED, pigment epithelial detachment; SD, standard deviation; SHRM, subretinal hyperreflective material; SRF, subretinal fluid.

Kruskal-Wallis test and Dunn pairwise test were used. ^1^ Values are significantly different from type 1 MNV; ^2^ Values are significantly different from type 2 MNV; ^3^ Values are significantly different from type 3 MNV.
