# Supplementary material for: Artificial intelligence-based analysis of retinal fluid volume dynamics in neovascular age-related macular degeneration and association with vision and atrophy
Source: Eye (Lond). 2024 Oct 15;39(1):154–61. doi: 10.1038/s41433-024-03399-1 (PMC11732971; doi:10.1038/s41433-024-03399-1)
Supplement: Supplementary file 5 — Supplemental Table 4. Distribution of ICF, SHRM, SRF and PED in the ETDRS Grid for Monthly Treated Eyes at Main Time Points. [file 41433_2024_3399_MOESM5_ESM.docx]

**Supplemental Table 4. Distribution of ICF, SHRM, SRF and PED in the ETDRS Grid for Monthly Treated Eyes at Main Time Points.**

|  |  | Mean volume distribution (in nL/mm2) per ETDRS grid region area (SE), nL | | | | | | |
| --- | --- | --- | --- | --- | --- | --- | --- | --- |
| Feature | ETDRS grid circles | Baseline | Month 1 | Month 3 | Month 6 | Month 12 | Month 18 | Month 24 |
| ICF | Central 1 mm | 46 (3) | 4 (1) | 4 (1) | 3 (1) | 3 (1) | 4 (1) | 3 (1) |
|  | Central 3 mm | 16 (1) | 2 (0) | 2 (0) | 1 (0) | 1 (0) | 2 (0) | 2 (0) |
|  | Central 6 mm | 5 (0) | 1 (0) | 1 (0) | 1 (0) | 1 (0) | 1 (0) | 1 (0) |
| SHRM | Central 1 mm | 74 (3) | 17 (2) | 9 (1) | 7 (1) | 5 (1) | 5 (1) | 5 (1) |
|  | Central 3 mm | 38 (2) | 9 (1) | 5 (1) | 5 (1) | 4 (1) | 4 (0) | 5 (1) |
|  | Central 6 mm | 13 (1) | 3 (0) | 2 (0) | 2 (0) | 2 (0) | 2 (0) | 2 (0) |
| SRF | Central 1 mm | 40 (3) | 17 (1) | 9 (1) | 9 (1) | 8 (1) | 8 (1) | 8 (1) |
|  | Central 3 mm | 36 (2) | 11 (1) | 6 (1) | 6 (1) | 6 (1) | 6 (1) | 6 (1) |
|  | Central 6 mm | 26 (1) | 5 (0) | 3 (0) | 3 (0) | 3 (0) | 3 (0) | 3 (0) |
| PED | Central 1 mm | 56 (4) | 33 (2) | 29 (2) | 28 (2) | 26 (2) | 25 (2) | 25 (2) |
|  | Central 3 mm | 35 (2) | 19 (1) | 17 (1) | 15 (1) | 14 (1) | 14 (1) | 14 (1) |
|  | Central 6 mm | 12 (1) | 6 (0) | 5 (0) | 5 (0) | 5 (0) | 5 (0) | 4 (0) |

Abbreviations: CFRV, cyst-free retinal volume; ETDRS, Early Treatment Diabetic Retinopathy Study; ICF, intraretinal cystoid fluid; PED, pigment epithelial detachment; nL, nanoliter; SE, standard error; SHRM, subretinal hyperreflective material; SRF, subretinal fluid.
