# Supplementary material for: Artificial intelligence-based analysis of retinal fluid volume dynamics in neovascular age-related macular degeneration and association with vision and atrophy
Source: Eye (Lond). 2024 Oct 15;39(1):154–61. doi: 10.1038/s41433-024-03399-1 (PMC11732971; doi:10.1038/s41433-024-03399-1)
Supplement: Supplementary file 6 — Supplemental Table 5. Multilinear Regression Model Estimates Associations of Every Additional 100 Nanoliter of Feature Volumes Distributed in ETDRS Grid at Main Time Points on BCVA at Month 24. [file 41433_2024_3399_MOESM6_ESM.docx]

**Supplemental Table 5. Multilinear Regression Model Estimates Associations of Every Additional 100 Nanoliter of Feature Volumes Distributed in ETDRS Grid at Main Time Points on BCVA at Month 24.**

| Feature | ETDRS grid | Mean coefficient (SE) | | | | |
| --- | --- | --- | --- | --- | --- | --- |
|  |  | Baseline (n = 929) | Month 1 (n = 921) | Month 3 (n = 918) | Month 12 (n = 915) | Month 18 (n = 899) |
| ICF | 1 mm | -5.5 (-7.6, -3.3) *** | -10.5 (-22, 0.7) * | -10.1 (-21.9, 1.6) | -13.8 (-20.6, -6.9) *** | -9.2 (-16, -2.5) ** |
|  | 3 mm | -1.7 (-2.5, -0.6) *** | -1.7 (-5.2, 1.4) | -1.3 (-4.6, 2.2) | -2.7 (-4.4, -0.9) ** | -2.2 (-4.2, -0.3) * |
|  | 6 mm | -1.4 (-2.1, -0.6) *** | -1 (-4, 1.1) | -1.3 (-3.7, 0.9) | -1.9 (-3.2, -0.7) ** | -2.4 (-4, -0.8) ** |
| SHRM | 1 mm | -3.5 (-5.4, -1.3) ** | -9.9 (-14.9, -5.2) *** | -12.5 (-20.3, -7.6) *** | -13.5 (-19.7, -7.3) *** | -17.1 (-25.5, -8.7) *** |
|  | 3 mm | -1.2 (-1.6, -0.7) *** | -2.7 (-3.9, -1.6) *** | -3.4 (-4.8, -2.4) *** | -3.4 (-4.5, -2.3) *** | -5.2 (-6.6, -3.8) *** |
|  | 6 mm | -0.9 (-1.3, -0.6) *** | -1.9 (-2.7, -1.2) *** | -1.8 (-3.1, -0.9) *** | -1.4 (-2, -0.9) *** | -3.2 (-4, -2.4) *** |
| SRF | 1 mm | -2.5 (-5.5, -0.1) * | 2 (-2.8, 6.0) | 3.4 (-3.7, 9.1) | 7.7 (2.5, 12.8) ** | 8.6 (3.1, 14) ** |
|  | 3 mm | -0.4 (-0.9, 0.1) * | -0.3 (-1.2, 0.6) | -0.1 (-1.5, 1.2) | 1.5 (0.5, 2.6) ** | 1.3 (0.4, 2.3) ** |
|  | 6 mm | -0.1 (-0.3, 0) | -0.3 (-0.7, 0.1) * | -0.3 (-0.9, 0.2) | 0.5 (0, 1.1) * | 0.3 (-0.1, 0.8) |
| PED | 1 mm | -0.4 (-2.7, 1.2) | 1.8 (-1.4, 4.2) | 3.7 (1.1, 6.3) * | 6.5 (3.4, 9.5) *** | 7.9 (4.5, 11.2) *** |
|  | 3 mm | -0.2 (-0.6, 0.2) | 0.1 (-0.7, 0.7) | 0.6 (0, 1.2) | 0.6 (-0.1, 1.3) | 0.9 (0.2, 1.6) * |
|  | 6 mm | -0.1 (-0.4, 0.1) | 0.2 (-0.5, 0.7) | 0.4 (-0.2, 0.9) | 0.1 (-0.4, 0.6) | 0.4 (-0.1, 0.9) |
| CFRV | 1 mm | -5.1 (-8.0, -2.5) *** | -6.6 (-10.4, -2.7) *** | -5.9 (-9.8, -1.9) ** | -5.4 (-9.2, -1.7) ** | -5.9 (-9.5, -2.2) ** |
|  | 3 mm | 0 (-0.5, 0.4) | -0.2 (-0.8, 0.4) | -0.3 (-0.9, 0.3) | -0.1 (-0.7, 0.5) | 0 (-0.6, 0.6) |
|  | 6 mm | 0.1 (0, 0.3) | 0.1 (-0.1, 0.3) | 0.1 (-0.1, 0.3) | 0.2 (-0.1, 0.4) | 0.1 (-0.1, 0.3) |

Abbreviations: BCVA, best-corrected visual acuity; CFRV, cyst-free retinal volume; CI, confidence interval; ETDRS, Early Treatment Diabetic Retinopathy Study; ICF, intraretinal cystoid fluid; nL, nanoliter; PED, pigment epithelial detachment; SHRM, subretinal hyperreflective material; SRF, subretinal fluid.

Data shown in mean ETDRS letters (95% CI).

* p < 0.05, ** p <0.01, *** p < 0.001.
