# Supplementary material for: Artificial intelligence-based analysis of retinal fluid volume dynamics in neovascular age-related macular degeneration and association with vision and atrophy
Source: Eye (Lond). 2024 Oct 15;39(1):154–61. doi: 10.1038/s41433-024-03399-1 (PMC11732971; doi:10.1038/s41433-024-03399-1)
Supplement: Supplementary file 7 — Supplemental Table 6. Logistic Regression Model Estimates Associations of Every Additional 100 nL of Feature Volumes in the Central 6 mm (diameter) Grid at Main Time Points on MA Development at Month [file 41433_2024_3399_MOESM7_ESM.docx]

# Supplemental Table 6. Logistic Regression Model Estimates Associations of Every Additional 100 nL of Feature Volumes in the Central 6 mm (diameter) Grid at Main Time Points on MA Development at Month 24.

| Feature | Baseline (n = 671) | | Month 1 (n = 645) | | Month 3 (n = 604) | | Month 12 (n = 541) | | Month 18 (n = 489) | |
| --- | --- | --- | --- | --- | --- | --- | --- | --- | --- | --- |
|  | OR (95% CI) | P value | OR (95% CI) | P value | OR (95% CI) | P value | OR (95% CI) | P value | OR (95% CI) | P value |
| ICF | 1.10 (1.01, 1.20) | 0.022 | 1.01 (0.82, 1.25) | 0.914 | 1.14 (0.91, 1.44) | 0.254 | 0.85 (0.44, 1.64) | 0.630 | 0.82 (0.14, 4.88) | 0.831 |
| SHRM | 1.03 (0.98, 1.08) | 0.218 | 1.04 (0.93, 1.16) | 0.465 | 1.03 (0.88, 1.20) | 0.728 | 1.01 (0.92, 1.12) | 0.797 | 0.80 (0.32, 2.02) | 0.640 |
| SRF | 0.96 (0.93,0.98) | 0.002 | 0.99 (0.95, 1.04) | 0.704 | 0.93 (0.82, 1.06) | 0.296 | 0.89 (0.73, 1.09) | 0.245 | 0.95 (0.67, 1.35) | 0.774 |
| PED | 1.03 (1.00, 1.06) | 0.047 | 0.97 (0.90, 1.04) | 0.396 | 0.93 (0.83, 1.05) | 0.242 | 0.96 (0.83, 1.11) | 0.584 | 0.70 (0.37, 1.33) | 0.274 |
| CFRV | 1.02 (1.00, 1.05) | 0.077 | 1.02 (0.99, 1.05) | 0.265 | 1.01 (0.97, 1.05) | 0.588 | 0.99 (0.94, 1.05) | 0.788 | 0.97 (0.86, 1.09) | 0.599 |

Abbreviations: CFRV, cyst-free retinal volume; CI, confidence interval; ICF, intraretinal cystoid fluid; MA, macular atrophy; OR, odds ratio; PED, pigment epithelial detachment; SHRM, subretinal hyperreflective material; SRF, subretinal fluid.
