# Supplementary material for: Artificial intelligence-based analysis of retinal fluid volume dynamics in neovascular age-related macular degeneration and association with vision and atrophy
Source: Eye (Lond). 2024 Oct 15;39(1):154–61. doi: 10.1038/s41433-024-03399-1 (PMC11732971; doi:10.1038/s41433-024-03399-1)
Supplement: Supplementary file 8 — Supplemental Figure Legends [file 41433_2024_3399_MOESM8_ESM.docx]

**Supplemental Figure 1. Illustration of The Process to Generate Volumetric Features From Spectral-domain Optical Coherence Tomography Volume Scans**

The four pathological features were intraretinal cystoid fluid (in red), subretinal hyperreflective material (in cyan), subretinal fluid (in green) and pigment epithelial detachment (in blue). Neurosensory retina was defined as the retinal tissue between internal limiting membrane (outlined in purple) and retinal pigment epithelium (outlined in orange).

**Supplemental Figure 2. Feature Segmentation Performance**

Segmentation performance matrix illustrating the Dice coefficient, positive predictive value, sensitivity and specificity of intraretinal cystoid fluid (ICF), subretinal hyperreflective material (SHRM), subretinal fluid (SRF) and pigment epithelial detachment (PED).

**Supplemental Figure 3. Distribution of Volumetric Features in the Early Treatment Diabetic Retinopathy Study Grid.**

Mean volumes of intraretinal cystoid fluid (ICF), subretinal hyperreflective material (SHRM), subretinal fluid (SRF) and pigment epithelial detachment (PED) over 24 months in the A, central 1 mm Early Treatment Diabetic Retinopathy Study (ETDRS) circle, B, central 3 mm ETDRS circle and C, central 6 mm ETDRS circle. Error bars indicate standard error. D, Mean volume distribution (nL/mm^2^) of IRC, SHRM, SRF and PED per ETDRS grid region area of ICF, SHRM, SRF and PED at baseline and month 3.

**Supplemental Figure 4. Feature funneling: selection process for data used to develop the logistic regression models estimates associations of feature volumes on macular atrophy (MA) development at Month 12.**

Data used to develop the logistic regression models to estimate the associations of feature volumes with MA development at Month 12 and Month 24
