# Supplementary figures and images for: Artificial intelligence-based analysis of retinal fluid volume dynamics in neovascular age-related macular degeneration and association with vision and atrophy
Source: Eye (Lond). 2024 Oct 15;39(1):154–61. doi: 10.1038/s41433-024-03399-1 (PMC11732971; doi:10.1038/s41433-024-03399-1)

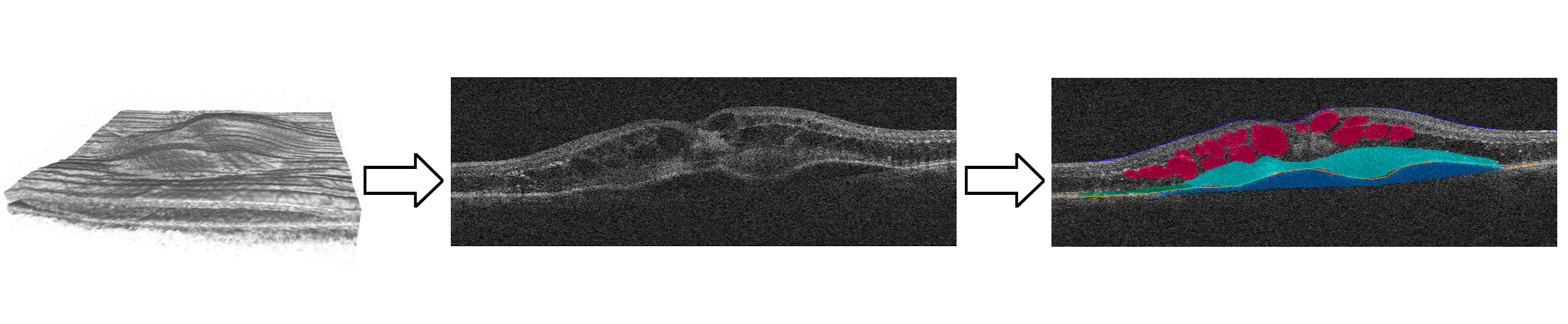

Supplement: Supplementary file 9 — Supplemental Figure 1. Illustration of The Process to Generate Volumetric Features From SD-OCT Volume Scans. [file 41433_2024_3399_MOESM9_ESM.tif]

Dice coefficient

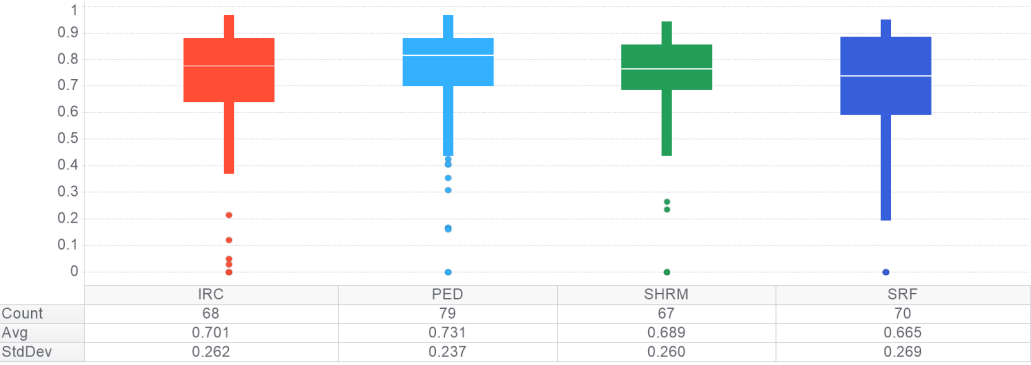

Sensitivity

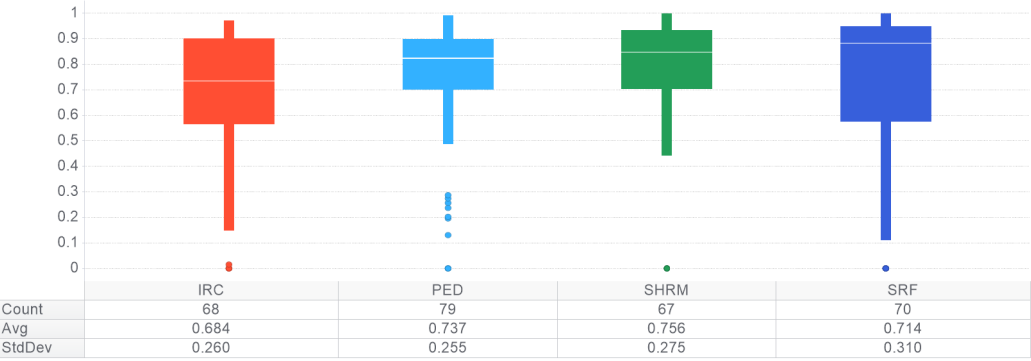

Volume difference in 100 nl

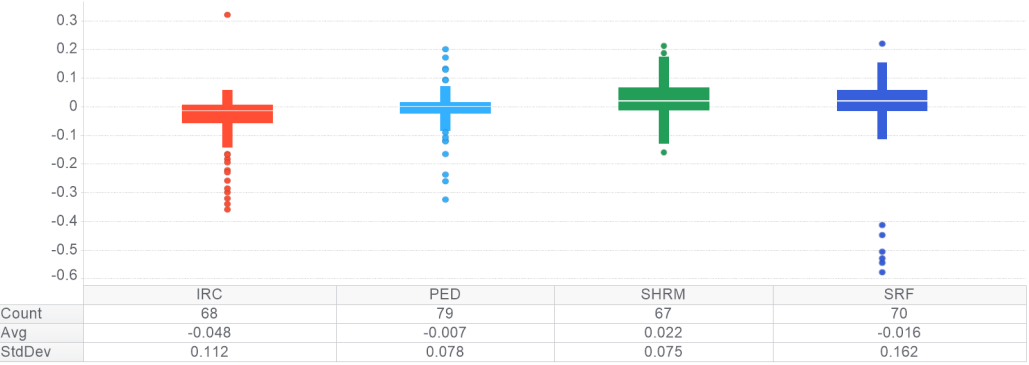

Specificity

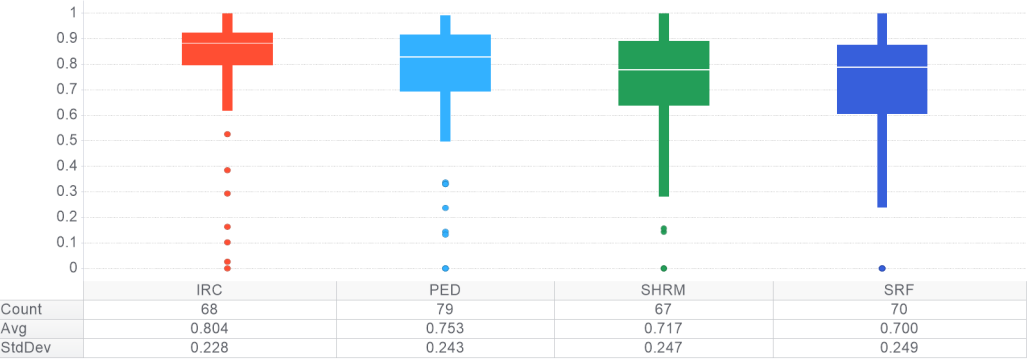

Supplement: Supplementary file 10 — Supplemental Figure 2. Feature Segmentation Performance. [file 41433_2024_3399_MOESM10_ESM.pdf]

A

Central 1mm circle

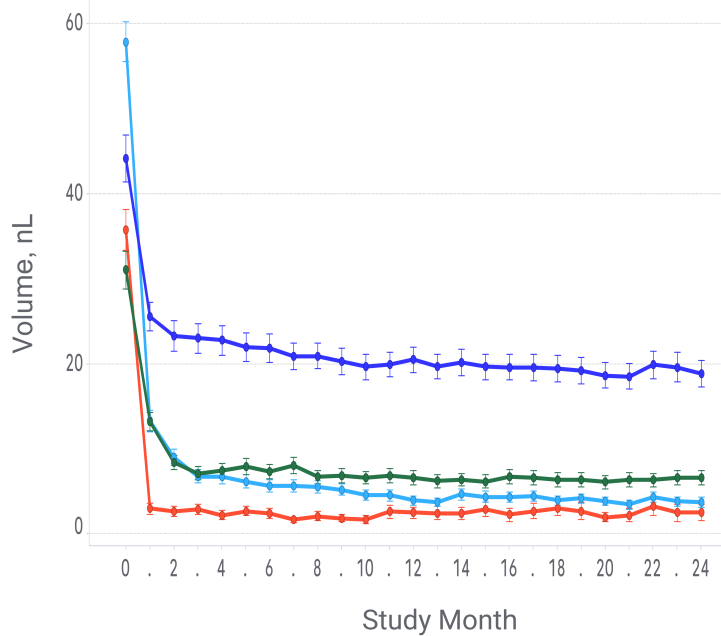

B

Central 3mm circle

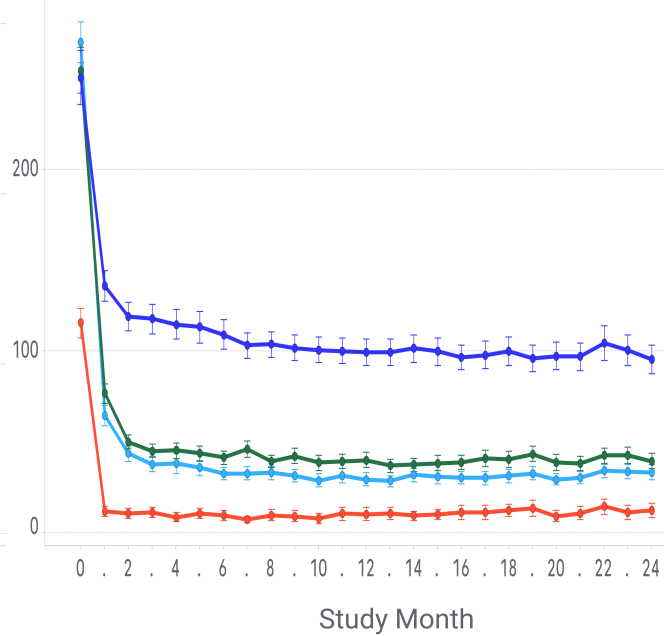

C

Central 6mm circle

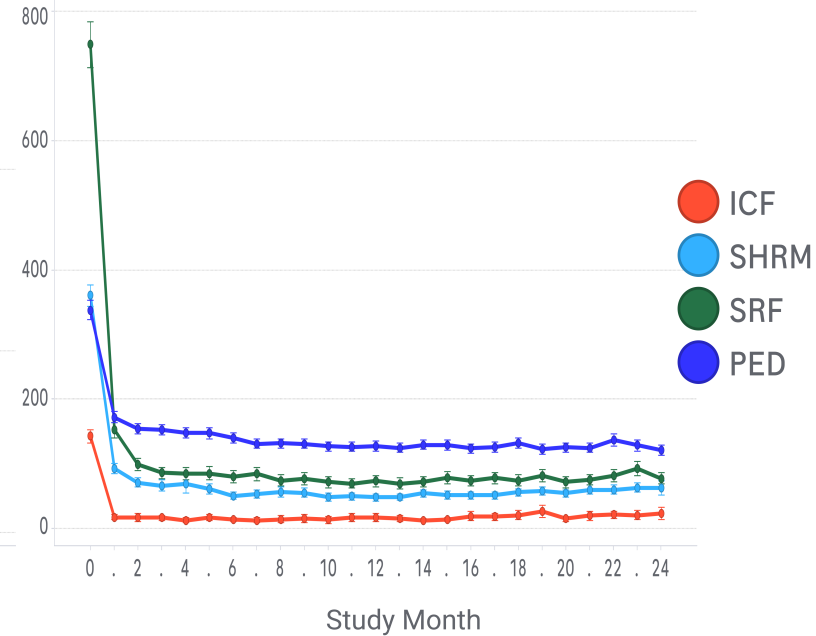

Supplement: Supplementary file 11 — Supplemental Figure 3. Distribution of Volumetric Features in the Early Treatment Diabetic Retinopathy Study Grid. [file 41433_2024_3399_MOESM11_ESM.pdf]

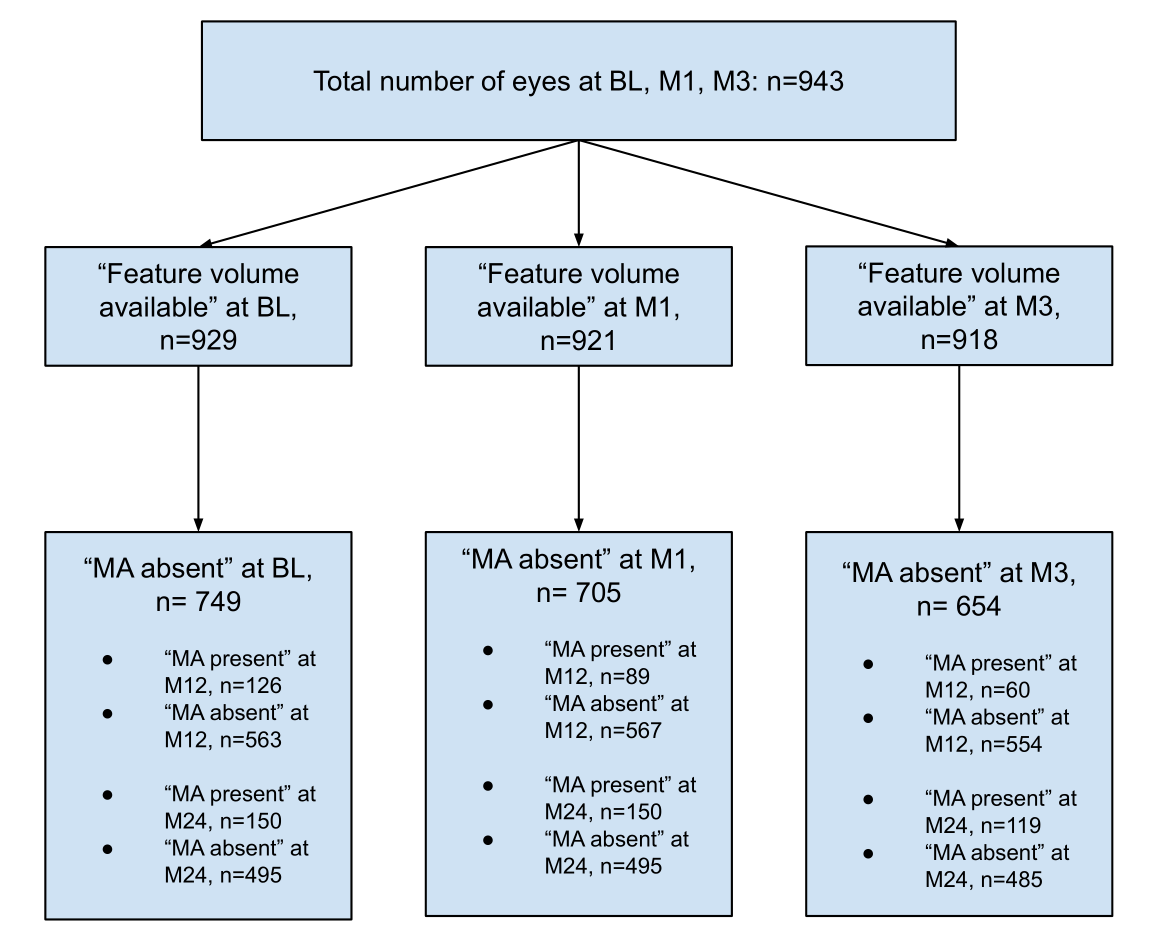

Supplement: Supplementary file 12 — Supplemental Figure 4. Feature funneling: selection process for data used to develop the logistic regression models estimates associations of feature volumes on MA development at Month 12. [file 41433_2024_3399_MOESM12_ESM.tif]
